# Supplementary material for: The Mitochondrial Ca2+ uniporter is a central regulator of interorganellar Ca2+ transfer and NFAT activation
Source: J Biol Chem. 2021 Sep 6;297(4):101174. doi: 10.1016/j.jbc.2021.101174 (PMC8496184; doi:10.1016/j.jbc.2021.101174)
Supplement: Figures S1–S9 — and Table S1 [file mmc1.pdf]

## ONLINE SUPPLEMENT

# The Mitochondrial $Ca^{2+}$ Uniporter is a Central Regulator of Interorganellar $Ca^{2+}$ Transfer and NFAT Activation

*By*


Ryan E. Yoast[1]†, Scott M. Emrich[1]†, Xuexin Zhang[1], Ping Xin[1,2,3], Vikas Arige[5], Trayambak Pathak[1,2,3], J. Cory Benson[1,2,3], Martin T. Johnson[1], Ahmed Emam Abdelnaby[1,2,3], Natalia Lakomski[1], Nadine Hempel[4], Jung Min Han[6], Geneviève Dupont[7], David I. Yule[5], James Sneyd[8], and Mohamed Trebak[1,2,3]*

*From*

[1]Department of Cellular and Molecular Physiology, the Pennsylvania State University College of Medicine, 500 University Drive, Hershey, PA 17033, USA

[2]Department of Pharmacology and Chemical Biology and [3]Vascular Medicine Institute, University of Pittsburgh School of Medicine, 200 Lothrop Street, Pittsburgh, PA 1526, USA

[4]Department of Pharmacology, The Pennsylvania State University College of Medicine, 500 University Drive, Hershey, PA 17033, USA

[5]Department of Pharmacology and Physiology, University of Rochester, 601 Elmwood Ave, Rochester, NY 14642 USA

[6]Laboratory of Biological Modeling, National Institute of Diabetes and Digestive and Kidney Diseases, National Institutes of Health, Bethesda, MD 20892, USA

[7]Unité de Chronobiologie Théorique, Université Libre de Bruxelles, CP231, Boulevard du Triomphe, 1050, Brussels, Belgium.

[8]Department of Mathematics, The University of Auckland, 38 Princes Street, Auckland, 1010, New Zealand.


**Running title:** MCU regulation of interorganellar $Ca^{2+}$ transfer and NFAT


**Key words**: MCU, mitochondria, calcium signaling, calcium oscillations, SOCE, CRAC channels, NFAT



*To whom correspondence should be addressed: trebakm@pitt.edu

†These authors contributed equally.

**Supplementary Figure Legends**:

**Supplementary Figure 1. MCU-KO in HeLa cells enhances cytosolic $Ca^{2+}$ upon thapsigargin stimulation.** Western blot documenting MCU protein knockout in MCU-KO HeLa cells compared to parental cells (**A**). Quantification of MCU protein band densitometry relative to GAPDH from three independent experiments analyzed using an unpaired t-test where (****$p < 0.0001$) (**A**). $Ca^{2+}$ measurements in HeLa parental cells and MCU-KO cells in response to stimulation with 2 µM thapsigargin (Tg) in $Ca^{2+}$-free buffer followed by restoration of 2 mM extracellular $Ca^{2+}$ to determine the magnitude of SOCE. Quantification and statistical analysis (Mann-Whitney test; ***$p < 0.001$) of SOCE (Δ SOCE) from at least three independent experiments are also shown (**B**).

**Supplementary Figure 2. MCU-KO cells have abrogated mitochondrial $Ca^{2+}$ uptake. (A)**, Simultaneous measurements of mitochondrial $Ca^{2+}$ uptake and membrane potential in permeabilized cell populations of wildtype and MCU-KO HEK293 cells. (**B-D**), Similar measurements to (**A**) in RBL-1, Jurkat and A20 cells, respectively. (**E-H**), Quantification of mitochondrial $Ca^{2+}$ uptake in wildtype and MCU-KO of HEK293, RBL-1, Jurkat and A20 cells, respectively. All experiments were performed at least three times and were statistically analyzed using a one-way ANOVA where (****$p < 0.0001$ and ***$p < 0.001$).

**Supplementary Figure 3. MCU-KO does not alter STIM1 protein expression. (A)**, Western blot for STIM1 and MCU proteins in wildtype and MCU-KO HEK293 cells. (**B**) Quantification of STIM1 band density relative to GAPDH from three independent experiments similar to (**A**). (**C, D**), Similar experiments to (**A, B**) in wildtype and MCU-KO Jurkat cells. Resulting protein expression data was statistically analyzed using a Mann-Whitney test where (ns, not significant $p>0.05$).

**Supplementary Figure 4. MCU-KO does not significantly affect mRNA expression of STIM/Orai isoforms.** QPCR on STIM and Orai isoforms from wildtype and MCU-KO cells of HEK293 and Jurkat. Results are representative of at least three independent experiments ± SEM.

**Supplementary Figure 5. MCU-KO promotes slow CDI of CRAC currents.** CDI recordings shown for all individual wildtype Jurkat cells.

**Supplementary Figure 6. MCU-KO promotes slow CDI of CRAC currents.** CDI recordings shown for all individual MCU-KO Jurkat cells.

**Supplementary Figure 7. Generation of tissue-specific MCU-KD mice.** $MCU^{flx/flx}$ $CD4^{Cre(+)}$ and $MCU^{flx/flx}$ $CD4^{Cre(-)}$ mice (**A**) and $MCU^{flx/flx}$ $MB1^{Cre(+)}$ and $MCU^{flx/flx}$ $MB1^{Cre(-)}$ mice (**B**) were identified by genotyping using specific primers as described in Methods.

**Supplementary Figure 8. MCU-KD has no effect on LPS-mediated B-cell proliferation. (A)**, Flow cytometry gating protocol used to assess B-cell proliferation. Intact and single B-cells are

gated based on SSC and FSC signals. Of these cells, live cells are gated based on APC-cy7 staining and these live cells are used to quantify B-cell populations with different CFSE fluorescence. (**B;** n=4), B-cell viability is not different between $MCU^{flx/flx}$ $MB1^{Cre(-)}$ and $MCU^{flx/flx}$ $MB1^{Cre(+)}$ populations stimulated with anti-IgM, anti-IgM + anti-CD40, anti-CD40 or lipopolysaccharides (LPS). (**C;** n=4) B-cell proliferation in response to 10 µg/mL LPS determined by CFSE staining is not significantly different between $MCU^{flx/flx}$ $MB1^{Cre(-)}$ and $MCU^{flx/flx}$ $MB1^{Cre(+)}$ populations. (**D;** n=4), Stimulation with 10 µg/mL anti-CD40 alone did not induce significant B-cell proliferation in either $MCU^{flx/flx}$ $MB1^{Cre(-)}$ or $MCU^{flx/flx}$ $MB1^{Cre(+)}$ cells. Proliferation and viability data was statistically analyzed using an unpaired t-test where (*$p < 0.05$, **$p < 0.01$, and ns, not significant $p>0.05$).

**Supplementary Figure 9**. **Model simulation of slow calcium-dependent inactivation of $I_{CRAC}$.** $Ca^{2+}$ influx through $I_{CRAC}$ (red curve plotted against the right-hand axis) and cytosolic $Ca^{2+}$ concentration (black curve plotted against the left-hand axis) changes over time in a modeled cell treated first with thapsigargin in nominally free external $Ca^{2+}$ until the ER was depleted. Upon restoration of external $Ca^{2+}$ at 100 min, $Ca^{2+}$ influx first increases quickly, then more slowly decays to a steady-state. The cytosolic $Ca^{2+}$ concentration increases more slowly before reaching steady-state at a lower value. These results are qualitatively consistent with Fig. 2A of(34).

**Supplementary Table 1.** List and source of reagents, antibodies, chemicals, primers, gRNA, cell lines and mice used in the study.

| Reagent type (species) or resource | Designation | Source or reference | Identifiers | Additional information |
|---|---|---|---|---|
| Genetic reagent *Mus musculus* | B6;129S-Mcutm1.1Jmol/J | Gift from Dr. John Elrod | | $MCU^{fl/fl}$ |
| Genetic reagent *Mus musculus* | B6(129X1)-Tg(Cd4-cre/ERT2)11Gnri /J | The Jackson Laboratory | Stock No: 022356 | $CD4CreER^{T2}$ |
| Genetic reagent *Mus musculus* | B6.C(Cg)-Cd79atm1(cre)Reth/EhobJ | The Jackson Laboratory | Stock No: 020505 | Mb1-Cre on C57BL/6 |
| Genetic reagent *Mus musculus* | $MCU^{fl/fl}$ $CD4CreER^{T2}$ | This paper | | |
| Genetic reagent *Mus musculus* | $MCU^{fl/fl}$ MB1Cre | This paper | | |
| Cell line (*Homo-sapiens*) | HCT116 (colon, epithelial) | ATCC | ATCC: CCL-247 | Male |
| Cell line (*Homo-sapiens*) | DLD1 (colon, epithelial) | ATCC | ATCC: CCL-221 | Male |
| Cell line (*Homo-sapiens*) | Jurkat, Clone E6-1 (Acute T cell leukemia) | ATCC | ATCC: TIB-152 | Male |
| Cell line (*Homo-sapiens*) | HEK293 (embryonic kidney) | ATCC | ATCC: CRL-1573 | Female |
| Cell line (*Homo-sapiens*) | HeLa (adenocarcinoma) | Gift from Dr. Suresh Joseph | | |
| Cell line (*Mus musculus*) | A20 (B cell lymphoblast) | ATCC | ATCC: TIB-208 | |
| Cell line (*Rattus norvegicus*) | RBL-1 (Basophilic leukemia) | ATCC | ATCC: CRL-1378 | |
| Cell line (*Homo-sapiens*) | HCT116 MCU KO (colon, epithelial) | This paper | | HCT116 MCU-KO cells were generated by the Trebak lab using CRISPR/Cas9 and are available upon request |
| Cell line (*Homo-sapiens*) | DLD1 MCU-KO (colon, epithelial) | This paper | | DLD1 MCU-KO cells were generated by the Trebak lab using CRISPR/Cas9 and are available upon request |
| Cell line (*Homo-sapiens*) | Jurkat, Clone E6-1 (Acute T cell leukemia) | This paper | | Jurkat MCU-KO cells were generated by the Trebak lab using CRISPR/Cas9 and are available upon request |

| | | | | |
|---|---|---|---|---|
| Cell line (*Homo-sapiens*) | HEK293 (embryonic kidney) | This paper | | HEK293 MCU-KO cells were generated by the Trebak lab using CRISPR/Cas9 and are available upon request |
| Cell line (*Homo-sapiens*) | HeLa (adenocarcinoma) | Gift from Dr. Suresh Joseph | | HeLa MCU-KO cells were generated by the Trebak lab using CRISPR/Cas9 and are available upon request |
| Cell line (*Mus musculus*) | A20 (B cell lymphoblast) | This paper | | A20 MCU-KO cells were generated by the Trebak lab using CRISPR/Cas9 and are available upon request |
| Cell line (*Rattus norvegicus*) | RBL-1 (Basophilic leukemia) | This paper | | RBL-1 MCU-KO cells were generated by the Trebak lab using CRISPR/Cas9 and are available upon request |
| Sequence-based reagent | MB1Common (1103) | The Jackson Laboratory | Genotyping primers | ACTGAGGCAGGAGGATTGG |
| Sequence-based reagent | MB1 WTForward (1104) | The Jackson Laboratory | Genotyping primers | CTCTTTACCTTCCAAGCACTGA |
| Sequence-based reagent | MB1 Mutant Forward (1105) | The Jackson Laboratory | Genotyping primers | CATTTTCGAGGGAGCTTCA |
| Sequence-based reagent | Positive Ctrl Forward (697) | The Jackson Laboratory | Genotyping primers | CTAGGCCACAGAATTGAAAGATCT |
| Sequence-based reagent | Positive Ctrl Rev (697) | The Jackson Laboratory | Genotyping primers | GTAGGTGGAAATTCTAGCATCATCC |
| Sequence-based reagent | $MCU^{fl/fl}$ Forward (357) | This paper | Genotyping primers | GAAGGCCTCCTGTTATGGAT |
| Sequence-based reagent | $MCU^{fl/fl}$ Reverse (358) | This paper | Genotyping primers | CCAGCTTGGTGAAGCCTGAT |
| Sequence-based reagent | CD4cre F (1249) | This paper | Genotyping primers | TCAAGGCCAGACTAGGCTGCCTAT |
| Sequence-based reagent | CD4cre R (1249) | This paper | Genotyping primers | TCTCTGTGGCTGGCAGTTTCTCCA |
| Sequence-based reagent | hSTIM1f (389) | Yoast et al. 2020 | qPCR primer | ATGCCAATGGTGATGTGGAT |
| Sequence-based reagent | hSTIM1r (390) | Yoast et al. 2020 | qPCR primer | CCATGGAAGGTGCTGTGTTT |
| Sequence-based reagent | hSTIM2f (1338) | This paper | qPCR primer | CGCTGGCAACAAATTGAGAAG |
| Sequence-based reagent | hSTIM2r (1339) | This paper | qPCR primer | CACCCAGCTGTGATCAGAATAA |
| Sequence-based reagent | hORAI1f (393) | Yoast et al. 2020 | qPCR primer | GATGAGCCTCAACGAGCACT |
| Sequence-based reagent | hORAI1r (394) | Yoast et al. 2020 | qPCR primer | ATTGCCACCATGGCGAAGC |
| Sequence-based reagent | hORAI2f (395) | Yoast et al. 2020 | qPCR primer | TGGCGGAAGCTCTACCTGAG |
| Sequence-based reagent | hORAI2r (396) | Yoast et al. 2020 | qPCR primer | CGGGTACTGGTACTGCGTC |

| | | | | |
|---|---|---|---|---|
| Sequence-based reagent | hORAI3f (397) | Yoast et al. 2020 | qPCR primer | CTGGAGAGTGACCACGAGTA |
| Sequence-based reagent | hORAI3r (398) | Yoast et al. 2020 | qPCR primer | TGGAGACCATGAGTGCAAAG |
| Sequence-based reagent | hGAPDHf (405) | Yoast et al. 2020 | qPCR primer | CCCTTCATTGACCTCAACTACA |
| Sequence-based reagent | hGAPDHr (405) | Yoast et al. 2020 | qPCR primer | ATGACAAGCTTCCCGTTCTC |
| Sequence-based reagent | hNONOf (238) | This paper | qPCR primer | TCCGAGGAGATACCAGTCGG |
| Sequence-based reagent | hNONOr (239) | This paper | qPCR primer | CCTGGGCCTCTCAACTTCGAT |
| Sequence-based reagent | hMCU gRNA1 | This paper | gRNA | AGAGTTGCTATCTATTCACC |
| Sequence-based reagent | hMCU gRNA2 | This paper | gRNA | ATCGCTTCCTGGCAGAATTT; |
| Sequence-based reagent | hMCU gRNA3 | This paper | gRNA | TGACAGCGTTCACGCCGGGA |
| Sequence-based reagent | msMCU gRNA1 | This paper | gRNA | GGGAGCCGCATATTGCAGTA |
| Sequence-based reagent | msMCU gRNA2 | This paper | gRNA | TTTACGACAACTGCAAGAGG |
| Sequence-based reagent | rMCU gRNA | This paper | gRNA | AAGCCTATCTCGGACTCCGT |
| Commercial assay or kit | cDNA Reverse Transcription Kit | Applied biosystems | Cat# 4368814 | |
| Commercial assay or kit | Guide-it Mutation Detection Kit | Clontech Laboratories | Cat# 631443 | |
| Commercial assay or kit | Pierce Rapid Gold BCA Protein Assay Kit | Thermo Fisher Scientific | Cat# A53225 | |
| Commercial assay or kit | EasySep Mouse $CD4^+$ T Cell Isolation Kit | STEMCELL Technologies | Cat# 19852 | |
| Commercial assay or kit | EasySep Mouse B Cell Isolation Kit | STEMCELL Technologies | Cat# 19854 | |
| Commercial assay or kit | Transcription factor staining kit | Tonbo Biosciences | Cat# TNB-0607-KIT | |
| Commercial assay or kit | RNeasy Mini Kit | Qiagen | Cat# 74104 | |
| Chemical compound, drug | Thapsigargin | Calbiochem | Cat# #586005 | |
| Chemical compound, drug | Ru360 | Calbiochem | Cat# 557440 | |
| Chemical compound, drug | Carbachol | Sigma-Aldrich | Cat #212385-100MG-M | |
| Chemical compound, drug | BAPTA-tetracesium Salt | Santa Cruz Biotechnology | Cat# 480436-84-8 | |

| | | | | |
|---|---|---|---|---|
| Chemical compound, drug | Antibiotic and Antimycotic | Thermo Fisher Scientific | Cat# 15240062 | |
| Chemical compound, drug | McCoy's 5A | Corning | Cat# 10-050CV | |
| Chemical compound, drug | RPMI-1640 | Corning | Cat# 10-040CV | |
| Chemical compound, drug | DMEM | Corning | Cat# 10-013-CV | |
| Chemical compound, drug | Lipofectamine 2000 | Thermo Fisher Scientific | Cat# 11668019 | |
| Chemical compound, drug | TrypLE | Thermo Fisher Scientific | Cat# 12605028 | |
| Chemical compound, drug | Glucose | Sigma-Aldrich | Cat# D9434 | |
| Chemical compound, drug | Puromycin | Gemini Bio Products | Cat# 02100552 | |
| Chemical compound, drug | Halt Protease Inhibitor Cocktail | Thermo Fisher Scientific | Cat# 78438 | |
| Chemical compound, drug | RIPA buffer | Sigma | Cat# R0278 | |
| Chemical compound, drug | NaCl | Fisher Scientific | Cat# S671 | |
| Chemical compound, drug | MOPS SDS running buffer | Thermo Fisher Scientific | Cat# NP0001 | |
| Chemical compound, drug | NuPAGE Transfer Buffer | Thermo Fisher Scientific | Cat# NP0006 | |
| Chemical compound, drug | KCl | Fisher Scientific | Cat# P217 | |
| Chemical compound, drug | $MgCl_2$ | Fisher Scientific | Cat# M33 | |
| Chemical compound, drug | $CaCl_2$ | Fisher Scientific | Cat# C614 | |
| Chemical compound, drug | HEPES | Fisher Scientific | Cat# BP310 | |
| Chemical compound, drug | Digitonin | EMD Millipore | Cat# 300410-250MG | |
| Chemical compound, drug | (Z)-4-Hydroxytamoxifen | Sigma-Aldrich | Cat# H7904-5MG | |
| Chemical compound, drug | NuPAGE Bis-Tris precast gels | Thermo Fisher Scientific | Cat# NP0321 | |
| Chemical compound, drug | Polyvinylidene difluoride membrane | Li-Core Biosciences | Cat# 88518 | |
| Chemical compound, drug | Odyssey Blocking Buffer (TBS) | Li-Core Biosciences | Cat# 937-50003 | |
| Chemical compound, drug | DNase I | Thermo Fisher Scientific | Cat# 18068-015 | |

| | | | | |
|---|---|---|---|---|
| Chemical compound, drug | Tween 20 | Fisher Scientific | Cat# BP337 | |
| Chemical compound, drug | FCCP | Cayman Chemical Company | Cat# 15218 | |
| Chemical compound, drug | ci-$IP_3$ | Tocris | Cat# 6210 | |
| Chemical compound, drug | EGTA-AM | Invitrogen | Cat# E1219 | |
| Software, algorithm | Image J | NIH | | https://imagej.net/ |
| Software, algorithm | Graphpad Prism 9 | GraphPad | | https://www.graphpad.com/scientific-software/prism/ |
| Software, algorithm | Clampfit 10.3 | Molecular Devices, LLC. | | https://www.moleculardevices.com/ |
| Software, algorithm | LAS X | Leica | | https://www.leica-microsystems.com/ |
| Software, algorithm | Image Studio Lite | | | https://www.licor.com/bio/image-studio-lite/download |
| Software, algorithm | Imaris for cell biologists | Oxford Instruments | | https://imaris.oxinst.com/products/imaris-for-cell-biologists |
| Software, algorithm | IDEAS for ImageStream | Luminex | | https://www.luminexcorp.com/imagestreamx-mk-ii/#software |
| Software, algorithm | FlowJo | BD biosciences | | |
| Antibody | IRDye 800CW Goat anti-Mouse | Li-Core Biosciences | Cat# 925-32210 | 1:10000 |
| Antibody | IRDye 800CW Donkey anti-Rabbit | Li-Core Biosciences | Cat# 925-32213 | 1:10000 |
| Antibody | α-MCU | Cell Signaling Technology | Cat# 14997S | 1:2000 |
| Antibody | α-STIM1 | Cell Signaling Technology | Cat# 4916S | 1:1000 |
| Antibody | α-NFAT1- Alexa Fluor 488 | Cell Signaling Technology | Cat# 14324S | 1:100 |
| Antibody | α-GAPDH | EMD Millipore | Cat# MAB374 | |
| Antibody | α-CD3 | Tonbo Biosciences | Cat# 70-0039-U100 | |
| Antibody | AffiniPure $F(ab')_2$ Fragment Goat Anti-Mouse IgM | Jackson ImmunoResearch | Cat# 115-006-075 | |
| Antibody | α-mouse CD40 | BioCell | Cat# BE0016-2 | |
| Other | Lipopolysaccharides from Escherichia coli O111:B4 | Millipore Sigma | Cat# L2630-10MG | |
| Other | Mito Tracker Deep red FM | Cell Signaling Technology | Cat# 8778S | |

| | | | | |
|---|---|---|---|---|
| Other | Dynabeads™ Mouse T-Activator CD3/CD28 | Thermo Fisher Scientific | Cat# 11456D | |
| Other | Fura-2 AM | Thermo Fisher Scientific | Cat# F1221 | |
| Other | Fura-FF | Cayman Chemical Company | Cat# 20415 | |
| Other | Cal520-AM | Bioquest | Cat# 21130 | |
| Other | CFSE | Thermo Fisher Scientific | Cat# C1157 | |
| Other | JC-1 | Thermo Fisher Scientific | Cat# T3168 | |
| Other | PowerUp SYBR Green Master Mix | Applied Biosystems | Cat# 100029285 | |
| Other | Recombinant Mouse IL-2 (Carrier-free) | Tonbo Biosciences | Cat# 21-8021-U020 | |

# Fig. S1

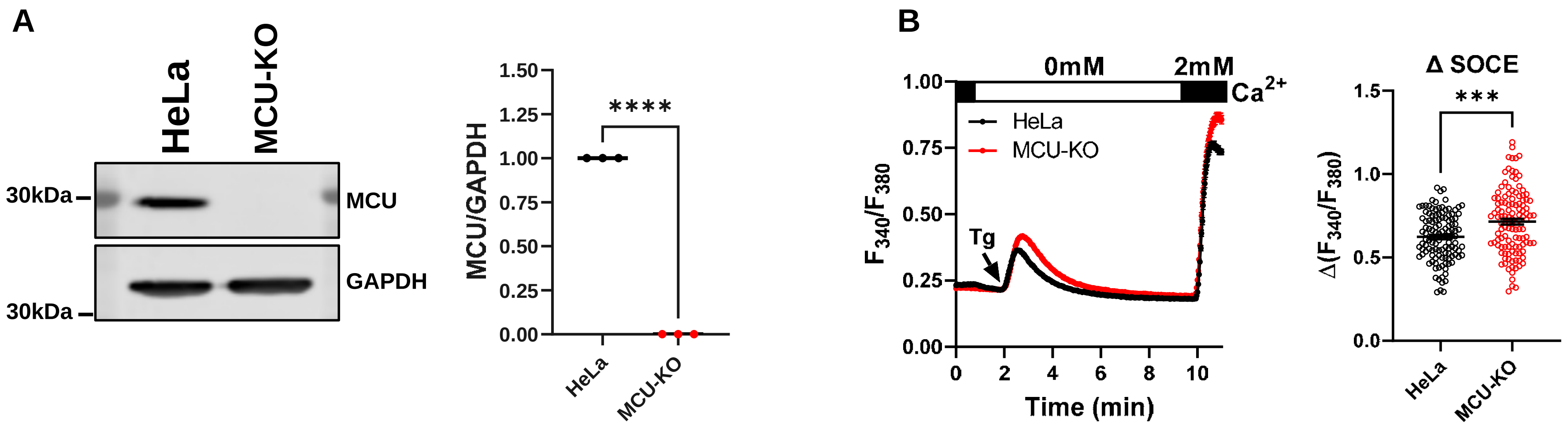

A
HeLa
MCU-KO
30kDa
MCU
GAPDH
30kDa
1.50
1.25
1.00
0.75
0.50
0.25
0.00
MCU/GAPDH
****
HeLa
MCU-KO
B
0mM
2mM
Ca2+
1.00
0.75
0.50
0.25
0.00
HeLa
MCU-KO
Tg
F340/F380
0
2
4
6
8
10
Time (min)
Δ SOCE
1.5
1.0
0.5
0.0
***
Δ(F340/F380)
HeLa
MCU-KO

Fig. S2

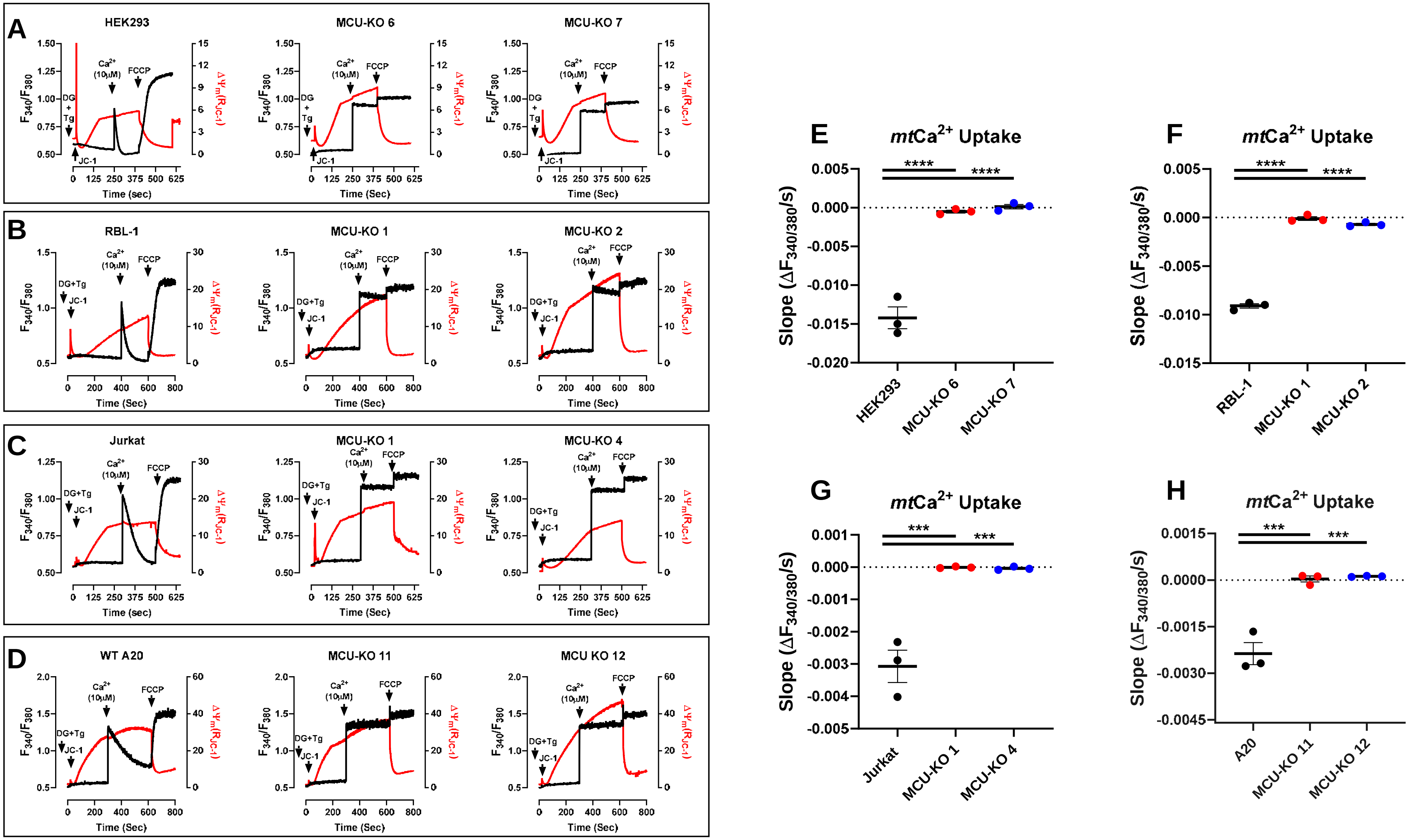

A
HEK293
MCU-KO 6
MCU-KO 7
B
RBL-1
MCU-KO 1
MCU-KO 2
C
Jurkat
MCU-KO 1
MCU-KO 4
D
WT A20
MCU-KO 11
MCU KO 12
DG+Tg
JC-1
Ca2+ (10μM)
FCCP
F340/F380
ΔΨm(RJC-1)
Time (sec)
Time (Sec)
E
F
G
H
mtCa2+ Uptake
Slope (ΔF340/380/s)
****
***
HEK293
RBL-1
A20

# Fig. S3

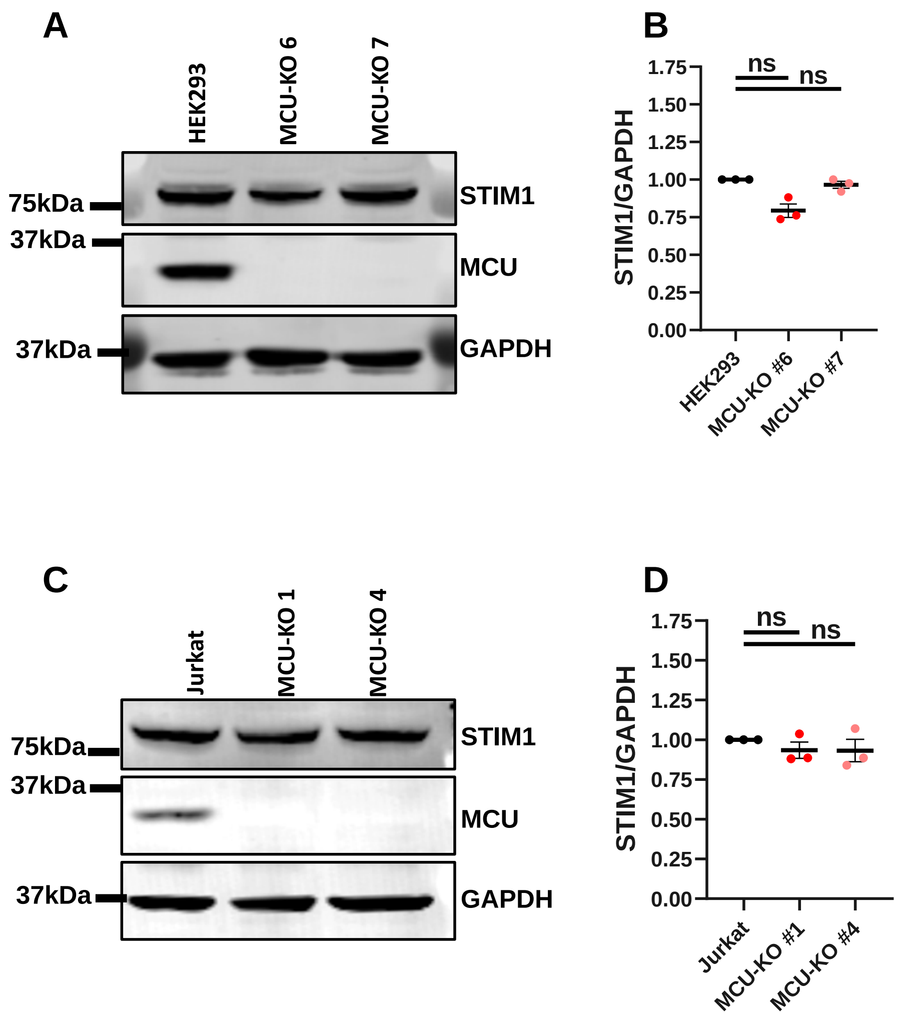
A
HEK293
MCU-KO 6
MCU-KO 7
75kDa
37kDa
37kDa
STIM1
MCU
GAPDH
B
STIM1/GAPDH
ns
ns
1.75
1.50
1.25
1.00
0.75
0.50
0.25
0.00
HEK293
MCU-KO #6
MCU-KO #7
C
Jurkat
MCU-KO 1
MCU-KO 4
75kDa
37kDa
37kDa
STIM1
MCU
GAPDH
D
STIM1/GAPDH
ns
ns
1.75
1.50
1.25
1.00
0.75
0.50
0.25
0.00
Jurkat
MCU-KO #1
MCU-KO #4

**Fig. S4**

| Cell Line | ORAI1 | ORAI2 | ORAI3 | STIM1 | STIM2 |
|---|---|---|---|---|---|
| **HEK293 MCU-KO 6** | 0.971±0.022 | 1.398±0.026 | 1.222±0.031 | 1.211±0.059 | 1.205±0.014 |
| **HEK293 MCU-KO 7** | 1.133±0.041 | 1.550±0.018 | 1.132±0.009 | 1.354±0.070 | 0.838±0.018 |
| **Jurkat MCU-KO 1** | 0.839±0.011 | 0.951±0.016 | 0.836±0.009 | 0.783±0.053 | 0.975±0.006 |
| **Jurkat MCU-KO 4** | 1.184±0.023 | 1.090±0.008 | 0.963±0.011 | 0.972±0.033 | 1.088±0.022 |

**Fig. S5**

WT Jurkat cells
1.2 mM EGTA; 0.66 mM $Ca^{2+}$

WT Jurkat cells
1.2 mM EGTA; 0.66 mM $Ca^{2+}$; Mito Cocktail

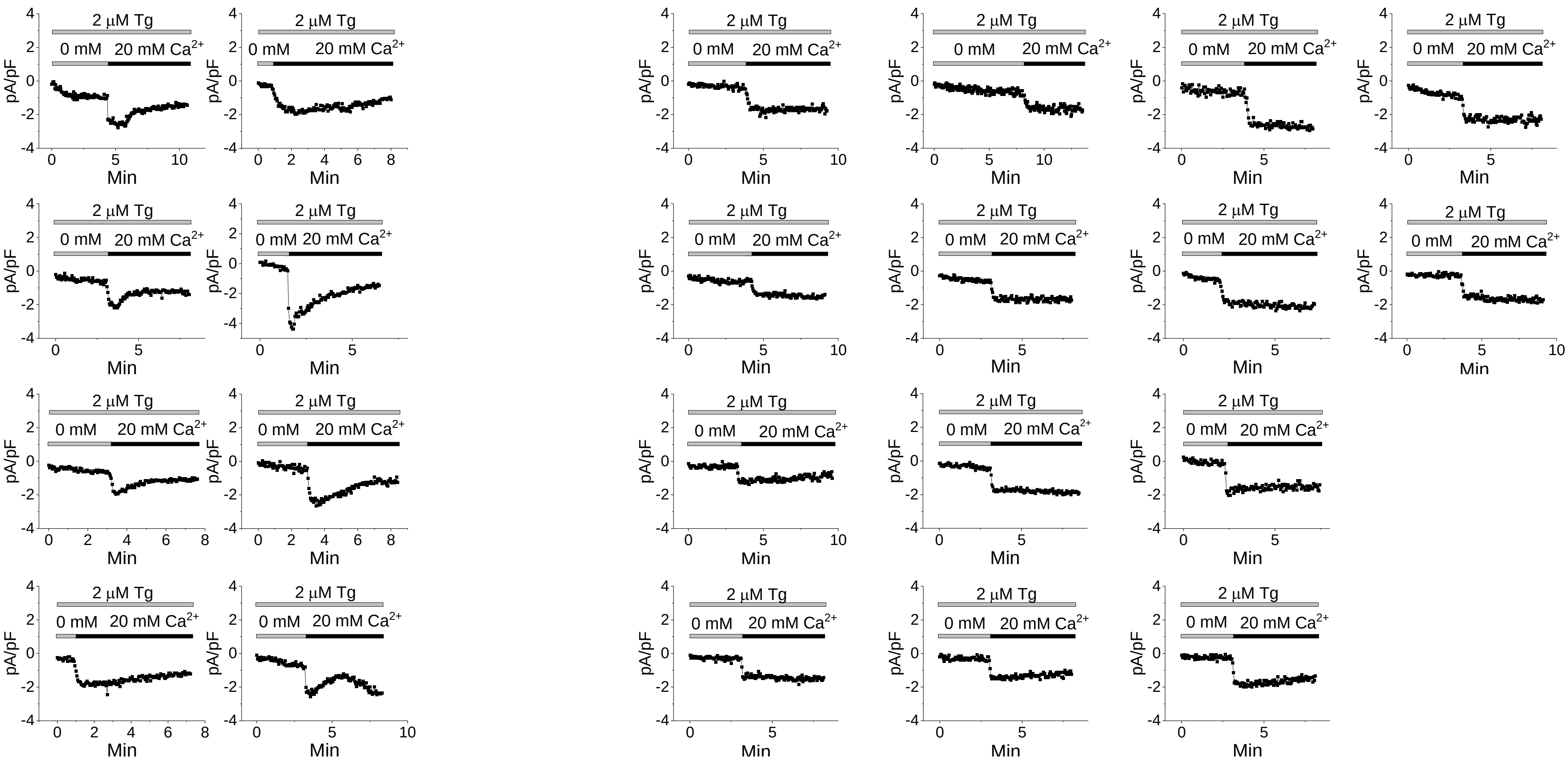

**Fig. S6**

MCU-KO (#1) Jurkat cells
1.2 mM EGTA; 0.66 mM $Ca^{2+}$

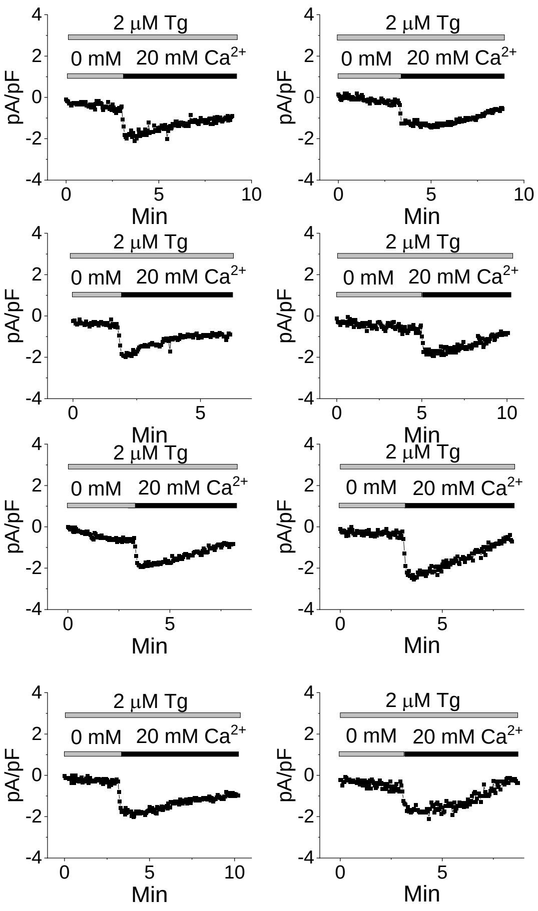


MCU-KO (#1) Jurkat cells
1.2 mM EGTA; 0.66 mM $Ca^{2+}$; Mito Cocktail

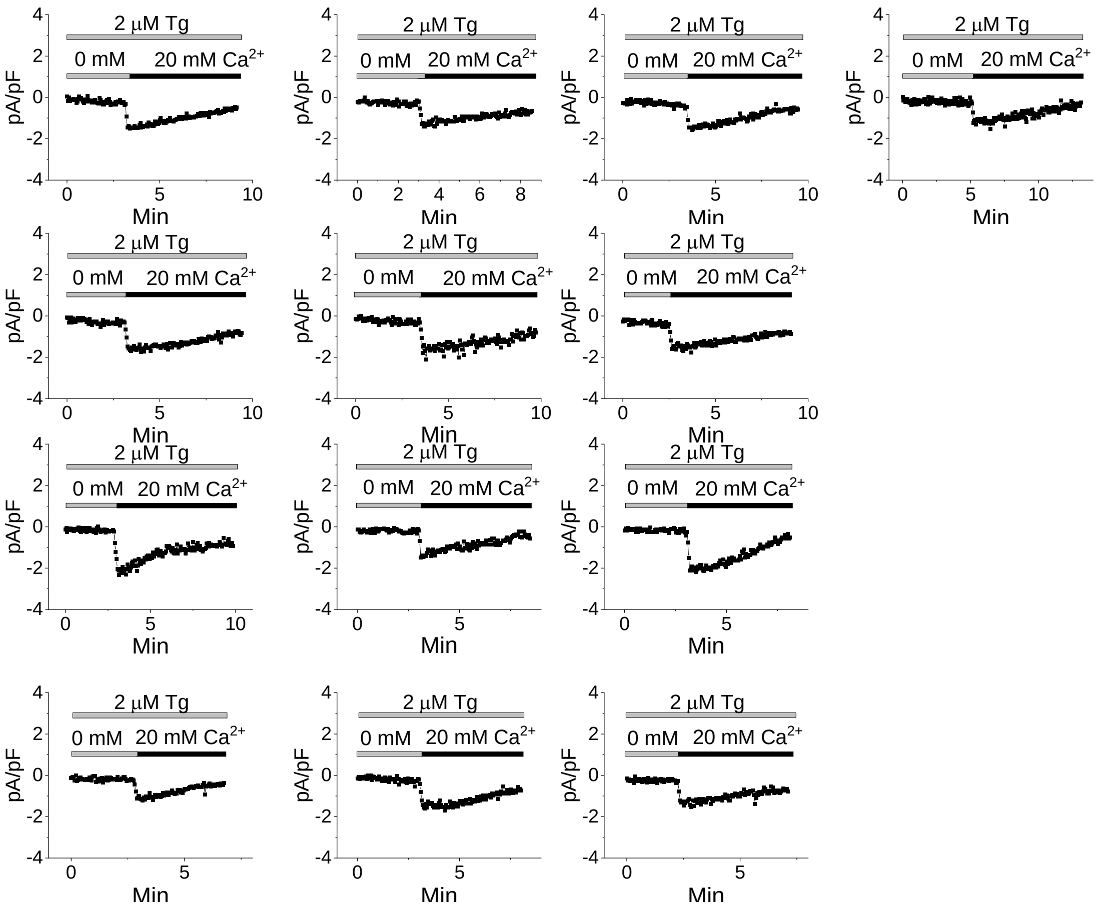

# Fig. S7

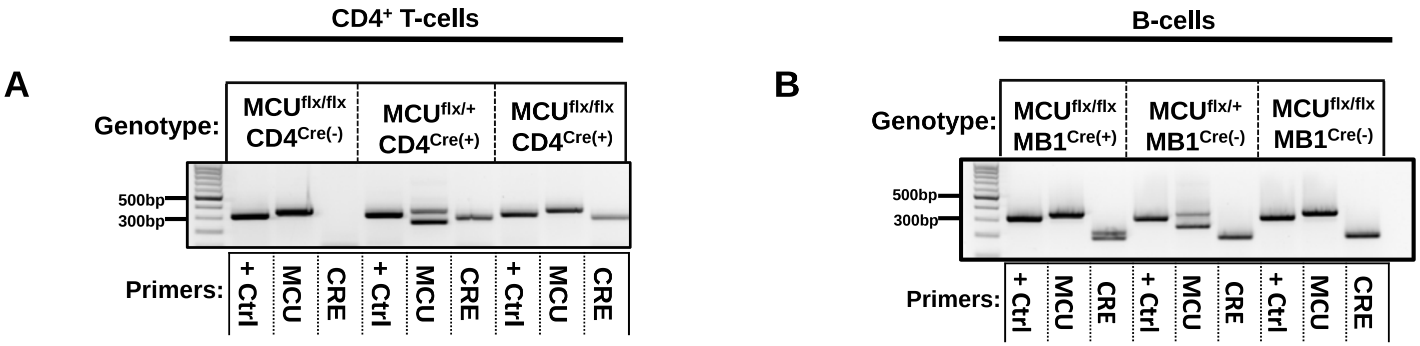

CD4+ T-cells
A
Genotype:
MCUflx/flx CD4Cre(-)
MCUflx/+ CD4Cre(+)
MCUflx/flx CD4Cre(+)
500bp
300bp
Primers:
+ Ctrl
MCU
CRE
+ Ctrl
MCU
CRE
+ Ctrl
MCU
CRE
B-cells
B
Genotype:
MCUflx/flx MB1Cre(+)
MCUflx/+ MB1Cre(-)
MCUflx/flx MB1Cre(-)
500bp
300bp
Primers:
+ Ctrl
MCU
CRE
+ Ctrl
MCU
CRE
+ Ctrl
MCU
CRE

Fig. S8

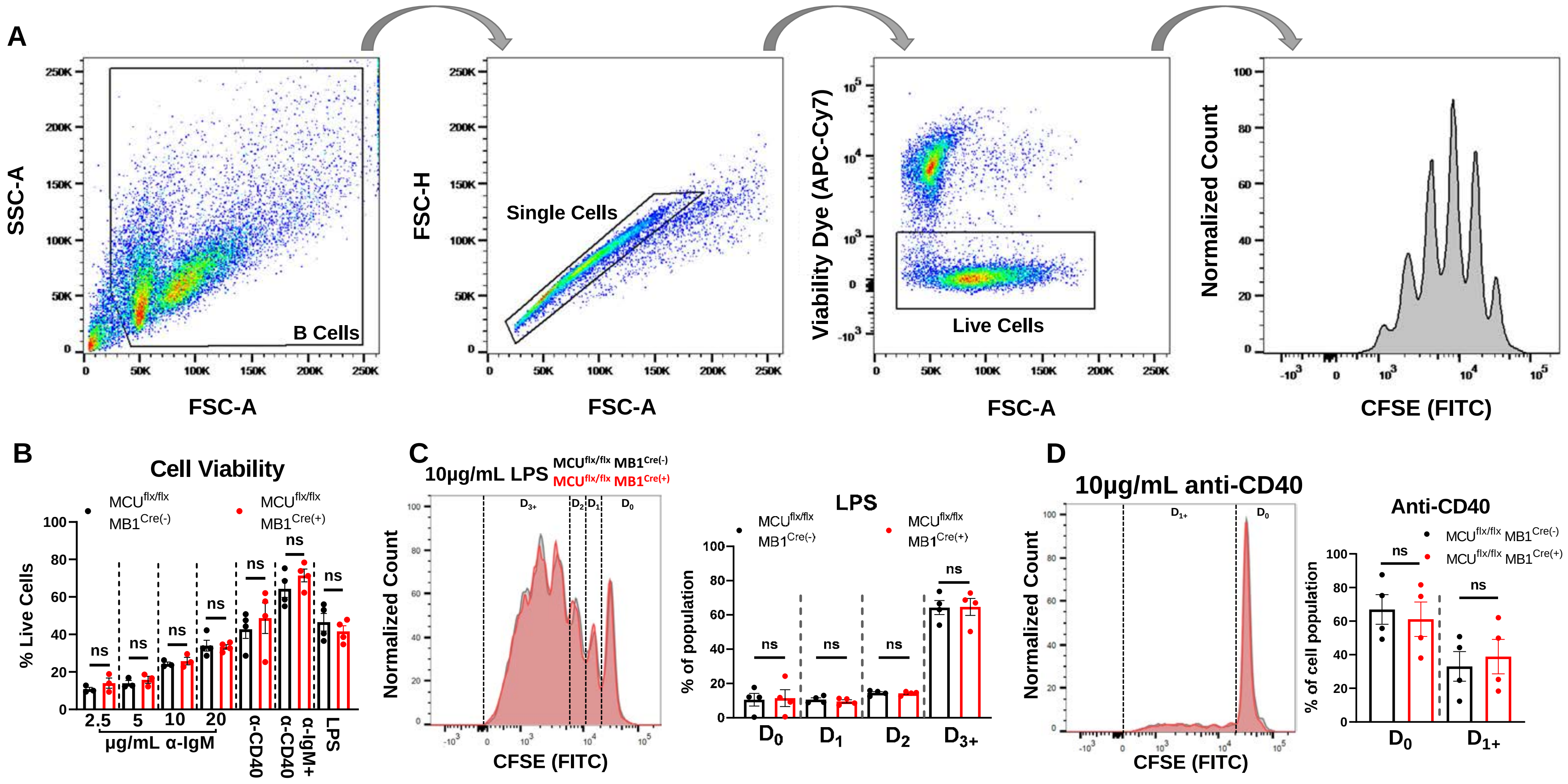
A
SSC-A
FSC-A
B Cells
FSC-H
Single Cells
Viability Dye (APC-Cy7)
Live Cells
Normalized Count
CFSE (FITC)
B
Cell Viability
MCUflx/flx MB1Cre(-)
MCUflx/flx MB1Cre(+)
% Live Cells
ns
2.5
5
10
20
µg/mL α-IgM
α-CD40
α-CD40 α-IgM+
LPS
C
10µg/mL LPS
MCUflx/flx MB1Cre(-)
MCUflx/flx MB1Cre(+)
D3+
D2
D1
D0
Normalized Count
CFSE (FITC)
LPS
% of population
D0
D1
D2
D3+
D
10µg/mL anti-CD40
D1+
D0
Normalized Count
CFSE (FITC)
Anti-CD40
% of cell population
D0
D1+

**Fig. S9**

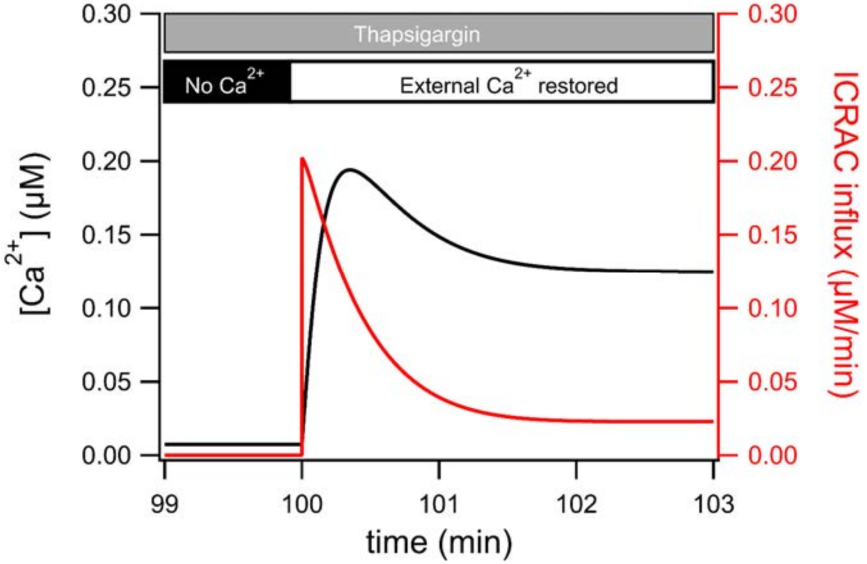
Thapsigargin
No Ca2+
External Ca2+ restored
0.30
0.25
0.20
0.15
0.10
0.05
0.00
[Ca2+] (µM)
ICRAC influx (µM/min)
99
100
101
102
103
time (min)
